# Mapping out a spectrum of the Chinese public’s discrimination toward the LGBT community: results from a national survey

**DOI:** 10.1186/s12889-020-08834-y

**Published:** 2020-05-12

**Authors:** Yuanyuan Wang, Zhishan Hu, Ke Peng, Joanne Rechdan, Yuan Yang, Lijuan Wu, Ying Xin, Jiahui Lin, Zhizhou Duan, Xuequan Zhu, Yi Feng, Shitao Chen, Jianjun Ou, Runsen Chen

**Affiliations:** 1 Department of Psychiatry & Mental Health Institute of the Second Xiangya Hospital, Central South University, National Clinical Research Centre on Mental Disorders (Xiangya), Hunan Medical Center for Mental Health, Changsha, 410011 Hunan China; 2 Division of Psychology, Faculty of Health and Life Sciences, De Montfort University, Leicester, UK; 3 State Key Laboratory of Cognitive Neuroscience and Learning, Beijing Normal University, Beijing, China; 4 The George Institute for Global Health, UNSW, Sydney, Australia; 5 School of Public Health, The University of Sydney, Sydney, Australia; 6 Department of Sociology, Peking University, Beijing, China; 7 Beijing LGBT Center, Beijing, China; 8 School of Health Sciences, Wuhan University, Wuhan, Hubei Province China; 9 The National Clinical Research Center for Mental Disorders, Beijing Key Laboratory of Mental Disorders & Advanced Innovation Center for Human Brain Protection, Beijing Anding Hospital, Capital Medical University, Beijing, China; 10 Mental Health Center, Central University of Finance and Economics, Beijing, China; 11 School of Psychology, Beijing Normal University, Beijing, China

**Keywords:** LGBT, China, Discrimination, Attitude

## Abstract

**Background:**

China has the world’s largest lesbian, gay, bisexual, and transgender (LGBT) population. This study assessed the discrimination experienced by LGBT individuals in China in a comprehensive way, covering discrimination perpetrated by family, media, medical services, religious communities, schools, social services, and in the workplace.

**Methods:**

The current study involved a national survey of 31 provinces and autonomous regions. Discrimination was measured both in terms of heterosexual participants’ attitudes towards LGBT individuals, and LGBT participants’ self-perceived discrimination. Pearson correlation analysis was performed to examine the difference between heterosexual participants’ attitudes towards LGBT individuals and LGBT participants’ self-perceived discrimination. Linear regression was used to investigate the association between gross domestic product per capita and discrimination.

**Results:**

Among 29,125 participants, 2066 (7.1%) identified as lesbian, 9491 (32.6%) as gay, 3441 (11.8%) as bisexual, 3195 (11.0%) as transgender, and 10,932 (37.5%) as heterosexual. Heterosexual people were generally friendly towards the LGBT community with a mean score of 21.9 (SD = 2.7, total scale score = 100) and the grand averaged score of self-perceived discrimination by LGBT participants was 49.9 (SD = 2.5). Self-perceived discrimination from family and social services is particularly severe. We created a series of provincial level choropleth maps showing heterosexual participants’ acceptance towards the LGBT community, and self-perceived discrimination reported by members of the LGBT community. We found that a higher level of economic development in provinces was associated with a decrease in discrimination, and we identified that every 100 thousand RMB increase in per capita GDP lead to a 6.4% decrease in discriminatory events perpetrated by heterosexuals.

**Conclusions:**

Chinese LGBT groups consistently experience discrimination in various aspects of their daily lives. The prevalence of this discrimination is associated with the economic development of the province in which it occurs. In order to reduce discrimination, it is important for future studies to discover the underlying reasons for discrimination against LGBT individuals in China.

## Background

As the country with the largest population, China is also home to the world’s largest lesbian, gay, bisexual and transgender (LGBT) community. In China, the LGBT community remains largely invisible in society, and its members consistently report experiencing barriers in their lives [1, 2]. In the first version of the Chinese Classification of Mental Disorders (CCMD; 1978), homosexuality was classified as sexual disorder [3]. Although the Chinese Society of Psychiatry no longer considers homosexuality a mental disorder as of 2001, the related stigma and discrimination against the LGBT community still remains in Chinese society [2]. Members of the LGBT community face social, cultural, and political discrimination, which may be why they remain a hidden sub-population [4]. Such discrimination marginalizes the LGBT community and can have an impact on their mental health and daily lives. There are approximately forty to seventy million LGBT individuals in China, and it is important to increase their social visibility, advocate for their rights, and reduce discrimination against them [5]. Researchers have paid considerable attention to investigating public discrimination against the LGBT community in a whole range of areas such as public service, schools, and employment [6]. However, there remains no comprehensive survey of discrimination against the LGBT community in China.

Previous studies have illustrated that economic development and modernization could increase tolerant attitudes towards sexual minority groups [7, 8]. However, compared with developed Western countries, modernization and economic development in China is still lacking. Moreover, unlike Western countries, Chinese people’s view of the LGBT community is strongly impacted by the distinctive Chinese cultural context. It is likely that LGBT individuals’ experiences pronounced negative feelings, psychological distress and perceive severe discrimination within the traditional Chinese cultural context [9]. In Chinese culture, there is an emphasis on obeying the ‘rules of nature’, due to the historical influence of Confucianism [10]. People are expected to conform to the gender identities/sexual orientations accepted by the vast majority, which is also a part of the Doctrine of the Mean (‘*Zhongyong’)*, which literally means average and ordinary in Confucianism [11]. Moreover, China is a collectivist culture and therefore Chinese people experience a great amount of influence from their family and society [2]. People in Chinese society emphasize family honor and dignity, and value maintaining ‘face’ (reputation) in social interactions. Being LGBT is still considered a form of shame for one’s family (‘losing face’) [12]. Having an LGBT family member could make the family the subject of vicious gossip, and stigmatize the family name [9, 13]. Parents of LGBT individuals will also be blamed for raising children who will fail to uphold their duty to carry on the family line. The Chinese government enacted a one-child policy in the 1970s in order to control the growing population, and the one-child policy was only recently replaced by a second child policy in the year of 2016 [14, 15]. Due to the influence of the one-child policy, the pressure of continuing the family line is extremely high for those in the one child only cohort. It is important to consider the Chinese context in order to provide a more in-depth understanding of the discrimination experienced by LGBT individuals across cultures.

Although there have been many public policies developed for improving the rights of stigmatized and marginalized LGBT individuals, they still face a great deal of discrimination in various contexts. In Japan, a qualitative study found that the discrimination experienced by the LGBT community was frequently evident in the development of laws and policies, employment, housing, healthcare, donating blood and education [16]. Similar results were identified in Korea. However, a positive change in attitudes toward LGBT people has been observed over the past two decades [17]. In China, it was reported that heterosexual people had an 11.1% rejection rate towards LGBT family members and a 2.1–4.1% rejection rate of social relationships with LGBT individuals [18]. While discrimination against the LGBT community in China has been studied [19], previous research rarely described the different levels of discrimination against the lesbian, gay, bisexual and transgender subgroups separately and comprehensively.

To develop appropriate strategies to reduce discrimination against the LGBT community, it is necessary to investigate the different aspects of discrimination faced by gay, lesbian, bisexual and transgender people in China as well as the geographic variation of discrimination. In the present research, we used a national sample of Chinese LGBT participants to examine the extent of self-perceived discrimination faced by each LGBT subgroup in different aspects of their daily lives, including interactions with family, medical services, religious communities, educational institutions, social services, in the media and in their workplace. Besides the self-perceived discrimination reported by LGBT participants, discriminatory attitudes towards members of the LGBT community were also measured by surveying a group of heterosexual participants. The association between seriousness of discrimination and economic development was additionally investigated.

## Method

### Sampling procedures

Between August 2015 and October 2015, we conducted a national survey via multiple sites. These sites included 24 community organizations working with sexual and gender minorities, educational institutions, LGBT social networks, and the United Nations Development Program’s social media. Participants were allowed to submit their responses in person, on paper, or online.

Multiple sampling strategies were applied in the current study including snowball, convenience, and respondent-driven sampling, with the majority of participants approached using online questionnaires. These strategies are proven to be sufficient for accessing stigmatized and discriminated populations [13, 20, 21]. We received 31,579 survey responses from all provinces, autonomous and special administrative regions. Of these, 29,125 were valid responses from LGBT and heterosexual individuals and were included in final analyses (which only included respondents from the 31 provinces of the Chinese mainland). More details about inclusion and exclusion criteria are given in the appendix. According to the categorization procedure described in Figure S1, the participants were categorized into five groups: lesbian, gay, bisexual, transgender, and heterosexual participants.

All participants provided informed consent before completing the survey. This study (secondary data analysis) was granted ethical approval by the Ethics Committee at Second Xiangya Hospital, Central South University.

### Measures

The questionnaire used in the current study was designed based on previous findings and expert consultation from the Beijing LGBT center [13, 20]. The questionnaire consisted of four parts: 1) Sample characteristics (demographic information); 2) Heterosexual participants’ self-reported acceptance towards members of the LGBT community; 3) LGBT participants’ reports of self-perceived discrimination; 4) Discrimination from public service providers (including educational institutions, public health institutions and the police) towards LGBT individuals. Each part of the survey contained several items. Rejection and discrimination were quantified as scores ranging from 0 to 100, with higher scores representing higher levels of rejection/discrimination. Further details about the measurements can be found in the Appendix.

### Data analysis and visualization

Chi-square tests were performed to examine the differences in basic characteristics between participants with different sexual orientations or gender identities. Heterosexual participants’ acceptance towards LGBT individuals was calculated and visualized in six national geographical (choropleth) maps. The grand averaged scores, indicating perceived discrimination against LGBT individuals across the seven settings, as well as general discrimination, were also calculated and visualized in choropleth maps of China.

Subsequently, we compared the results of the current study and those of a previous study. We examined similarities and differences in disclosure of sexual orientation/gender identity, perceived discrimination against LGBT individuals, heterosexual participants’ rejection and discrimination towards LGBT individuals, and heterosexual participants’ rejection towards LGBT individuals in relation to GDP. It should be noted that Tibet had the lowest sample size. The data from Tibet could therefore have severely undermined the correlation pattern. As such, we excluded this data from the correlation analysis.

In a recent study, RY Chua, KG Huang and M Jin [22] examined tolerance toward LGBT individuals among participants from 31 Chinese provinces. Pearson correlation analysis was performed to examine whether the discrimination measured in the current study was consistent with their results. Similar to RY Chua, KG Huang and M Jin [22], we standardized the general rejection scores among all provinces, and we added 3 to all of the provinces’ scores. In RY Chua, KG Huang and M Jin [22] study, the scores denoting tolerance towards LGBT individuals ranged from 0 to 5. To compare with the tolerance scores, we subtracted the general rejection scores from 5 to form tolerance scores, with larger scores representing higher tolerance towards the LGBT community.

Furthermore, we examined whether the heterosexual participants’ rejection (measured by general rejection scores) was consistent with the perceived discrimination reported by the LGBT participants (measured by general discrimination scores). The Pearson correlation between these two aspects was calculated. Furthermore, since data on the LGBT participants’ disclosure and perceived violent events was collected using two items across five scenarios (including family, school, medical service, workplace, and religion), it is worth exploring whether disclosure in certain environments was related to experiencing more violent events. For this purpose, we performed multivariate analyses after adjusting for age and education.

In addition, many studies have showed that higher economic growth is associated with higher social tolerance [8]. Therefore, we performed linear regression analyses to investigate how gross domestic product (GDP) per capita (http://www.stats.gov.cn/tjsj/ndsj/2016/indexch.htm) influences discrimination against members of the LGBT community (measured by general rejection scores).

## Results

### Basic characteristics of the sample

The sample size across the 31 provinces in mainland China for each group are displayed in Figure S2, and the basic characteristics of the sample of are listed in Table 1. Further details about the sample are provided in the appendix.

**Table 1:** Baseline socio-demographic characteristics of the participants

	Lesbian		Gay		Bisexual		Transgender		Heterosexual	
	(*n* = 2066)		(*n* = 9491)		(*n* = 3441)		(*n* = 3195)		(*n* = 10,932)	
	N	%	N	%	N	%	N	%	N	%
Age < 20	541	26.2	2380	25.1	1123	32.6	982	30.7	2720	24.9
Age 20–29	1343	65	5978	63	2117	61.5	1861	58.2	6940	63.5
Age 30–39	175	8.5	903	9.5	166	4.8	283	8.9	946	8.6
Age 40+	7	0.3	230	2.4	35	1	69	2.2	326	3
Han Chinese	1910	92.5	8818	92.9	3190	92.7	2946	92.2	10,194	93.3
Urban residency	2027	98.1	9014	95	3359	97.6	2952	92.4	10,415	95.3
Attended college or above	1253	60.7	5169	54.5	2218	64.5	1225	38.3	6206	56.8
Unemployed	198	9.6	996	10.5	265	7.7	450	14.1	1113	10.2
Having religious group	296	14.3	1672	17.6	484	14.1	705	22.1	1809	16.6
Having disability	9	0.4	95	1	26	0.8	41	1.3	–	–
Married:	51	2.5	436	4.6	164	4.8	228	7.1	–	–
Heterosexual Marriage	19	37.3	377	86.5	151	92.1	201	88.2	–	–
Cooperative marriage	30	58.8	52	11.9	13	7.9	23	10.1	–	–
Married oversea	2	3.9	7	1.6	0	0	4	1.8	–	–

### Provincial level friendly environments

**Fig. 1:**
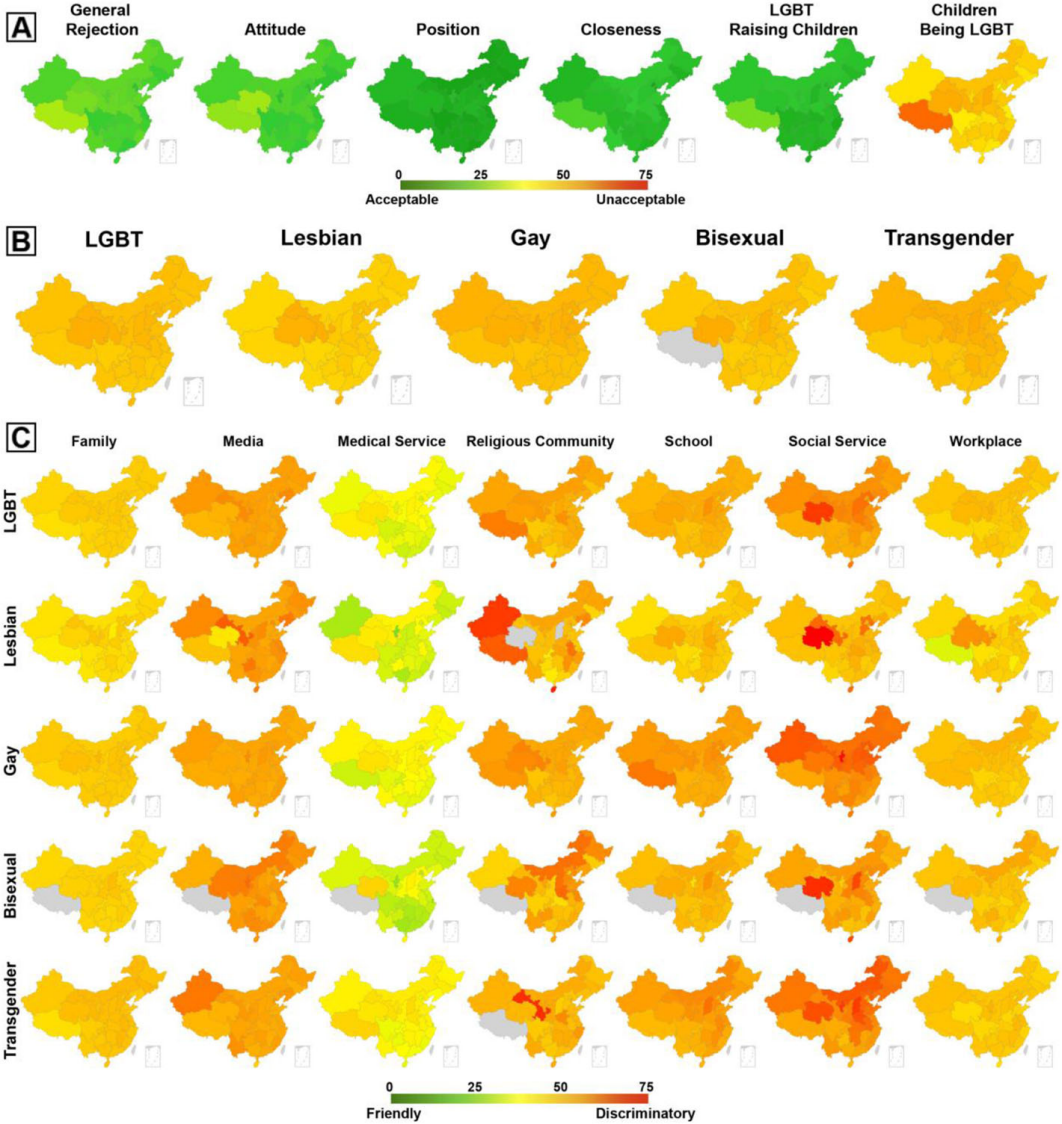
Choropleth maps (unit: percentage): **a** Heterosexual participants’ acceptance towards lesbian, gay, bisexual and transgender (LGBT) persons. The general rejection is the sum of the breakdown five dimensions assessment. **b** General discrimination against LGBT persons overall and by group. **c** Discrimination against LGBT persons in different environments

As in Fig. 1a, across the 31 provinces in mainland China, heterosexual participants showed tolerance towards the LGBT community with a mean *general rejection* score of 21.9 (SD = 2.7, total scale score = 100). A relatively higher level of rejection was found in the northwestern provinces of China (including Tibet, Qinghai, Shaanxi, and Gansu), and central China (Henan) (Mean = 25.8, SD = 2.6). In contrast, Shanghai, Tianjin, Beijing, Guangdong, Jiangsu, and Sichuan were found to be LGBT friendly, with scores under 20 (mean = 18.5, SD = 1.4). Of the five dimensions of acceptance assessed, heterosexual participants reported a low level of acceptance of having children who identify as LGBT (Mean = 46.4, SD = 5.5). There was a noticeably low overall acceptance rate in the westernmost region of China; Tibet had the lowest acceptance rate, followed by Qinghai, Shanxi, Gansu and Shaanxi (Mean = 55.2, SD = 4.5). In contrast, overall, acceptance of the position of LGBT persons (Mean = 11.3, SD = 1.8), getting close to LGBT persons (Mean = 15.7, SD = 2.6) LGBT persons raising children (Mean = 15.2, SD =2.9) was found to be high. Moderate-high acceptance level of general attitude towards LGBT was identified (Mean = 20.5, SD = 2.9). Furthermore, the distribution of general attitude shown in the map (Fig. 1a) was in line with the distribution of general rejection.

Meanwhile the perceived discrimination reported by LGBT participants was also visualized. As shown in Fig. 1b, the grand averaged score of self-perceived discriminations across seven environments was 49.9 (SD = 2.5). Gay men (Mean = 51.4, SD = 1.6) and transgender persons (Mean = 51.4, SD = 2.0) reported the highest rate of self-perceived discrimination, while less severe of discrimination was reported by lesbians (Mean = 47.8, SD =2.3). Consistent with the rejection of LGBT individuals reported by heterosexual people, a higher rate of self-perceived discrimination was more commonly reported by LGBT participants living in the north of China (Qinghai, Gansu, Shanxi, and Hebei), and Henan in central China (mean = 52.5, SD = 0.6).

We mapped perceived discrimination against LGBT persons separately for each of the seven environments. As shown in Fig. 1c, of the seven settings, the highest level of self-perceived discrimination was reported for social services (Mean = 57.1, SD = 5.7), and discrimination was higher in the north of China. LGBT participants also reported relatively higher levels of self-perceived discrimination in religious settings, with a mean score of 53.5, with a noticeably high incidence among lesbians living in Hainan (Mean = 70.6) and Xinjiang (Mean = 69.2), and transgender persons living in Gansu (Mean = 68.1). Self-perceived discrimination was generally lower in medical service settings (mean = 36.7, SD =3.8), with the exception of transgender participants, who reported an elevated rate (mean = 39.6, SD =2.7). In school settings, LGBT participants reported a moderate to high level of discriminatory behaviors by peers or teachers (mean = 52.9, SD =3.9), with a relatively lower level of discrimination found among the lesbian group (mean = 48.9, SD =3.1). LGBT participants reported less attention and biased reporting on their community by Chinese media (mean = 56.3, SD =2.7). In family (mean = 47.5, SD =2.3) and workplace settings (mean = 48.4, SD =3.1), a moderate rate of perceived discrimination was reported by LGBT participants.

#### The disclosure status of sexual orientation and gender identity of LGBT participants

The disclosure levels of the sexual minority groups are listed in Table 2. Lesbian participants had the highest rate of sexual orientation disclosure to family (70.1%), in school (67.3%) and the workplace (36.0%). In school, transgender individuals and gay men were found to be more likely to hide their gender identity and sexual orientation compared to lesbians and bisexual persons (χ2 = 440.5, df = 3, *p* < 0.01). Furthermore, gay men were less likely to report their sexual orientation in their workplace (χ2 = 116.0, df = 3, *p* < 0.01). Bisexual participants reported being more willing to disclose their sexual orientation when receiving medical services (χ2 = 116.0, df = 3, *p* < 0.01). Interestingly, transgender participants reported a higher disclosure rate when receiving social services (χ2 = 39.0, df = 3, *p* < 0.01). No statistically significant group differences were found regarding disclosure in religious settings.

**Table 2:** Disclosure in different Environments

	Lesbian		Gay		Bisexual		Transgender		Statistics		
	N	%	N	%	N	%	N	%	X^2^	df	p
Disclosure to family	1449	70.1	4589	48.4	1718	49.9	1516	47.5	347.1	3	<0.01
^a^Disclosure in school	1390	67.3	4238	44.7	1815	52.8	1314	41.2	440.5	3	<0.01
^b^Disclosure in workplace	412	36	1145	21.3	315	22.4	496	25.4	116	3	<0.01
^c^Disclosure to religious group	73	24.7	331	19.8	94	19.4	137	19.4	4.3	3	0.23
Disclosure in medical service	1555	75.3	7351	77.5	2783	80.9	2399	75.1	39	3	<0.01
Disclosure in social service	172	8.3	689	7.3	287	8.3	368	11.5	56.7	3	<0.01

a: all samples received education, b: all samples had employment experience, c: all samples had religious affiliations

#### Validation of heterosexuals’ rejection of the LGBT community

**Fig. 2:**
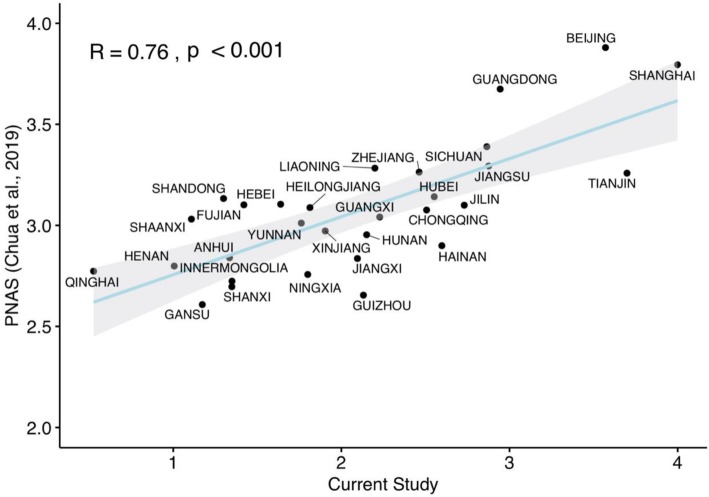
Consistency between results from the current study and Chua et al.’s [22] study

**Fig. 3:**
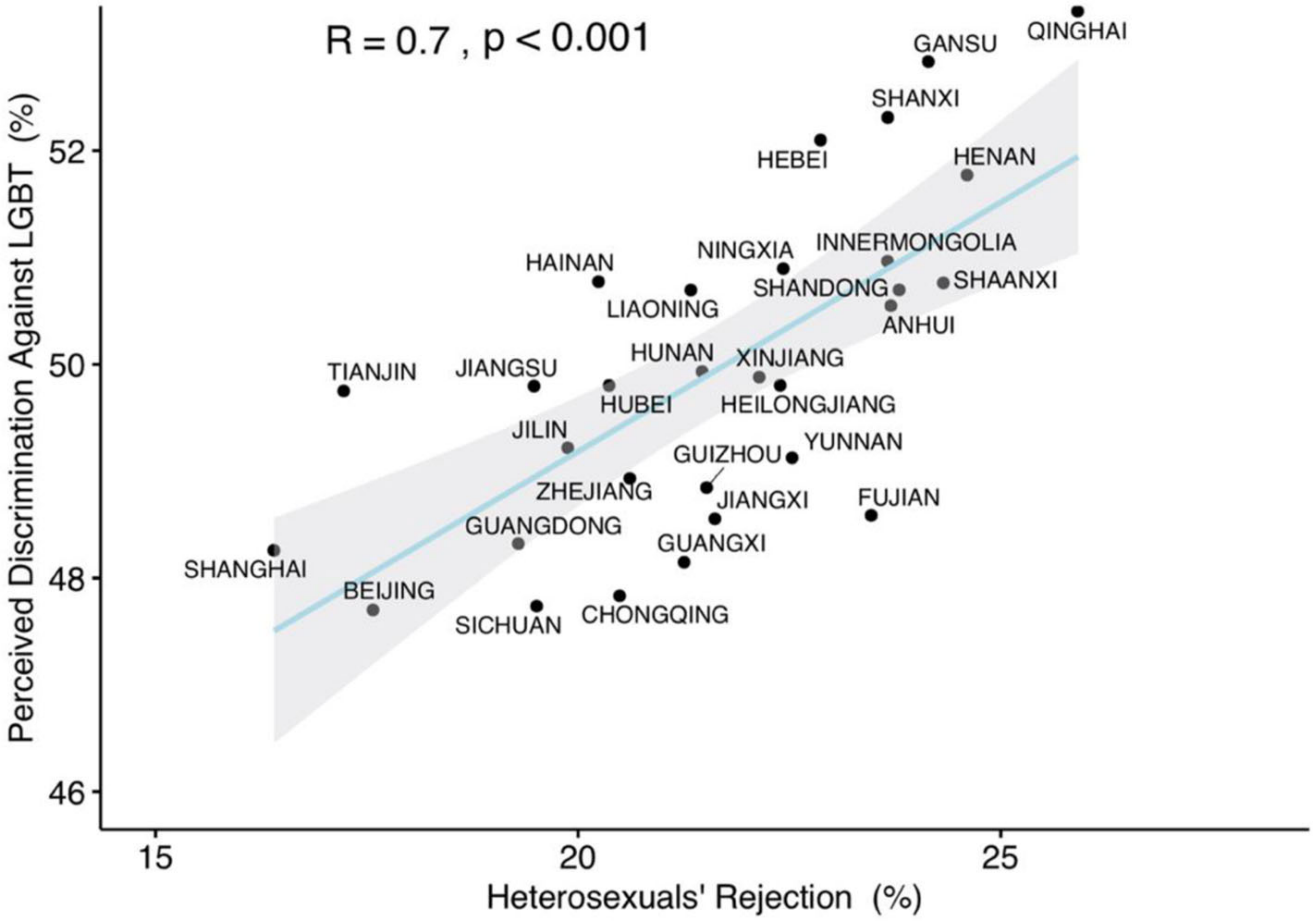
Consistency between heterosexuals’ rejection and perceived discrimination against LGBT persons

Heterosexual participants’ self-reported tolerance towards the LGBT community in the present study showed a correlation with that in RY Chua, KG Huang and M Jin [22] study (r = 0.76, *P* < 0.001) (see Fig. 2). Furthermore, in the current study, heterosexual participants rejection of the LGBT community was also correlated with LGBT participants’ self-perceived reports of discrimination (r = 0.70, *P* < 0.001) (see Fig. 3).

### Heterosexuals’ rejection versus GDP

**Fig. 4:**
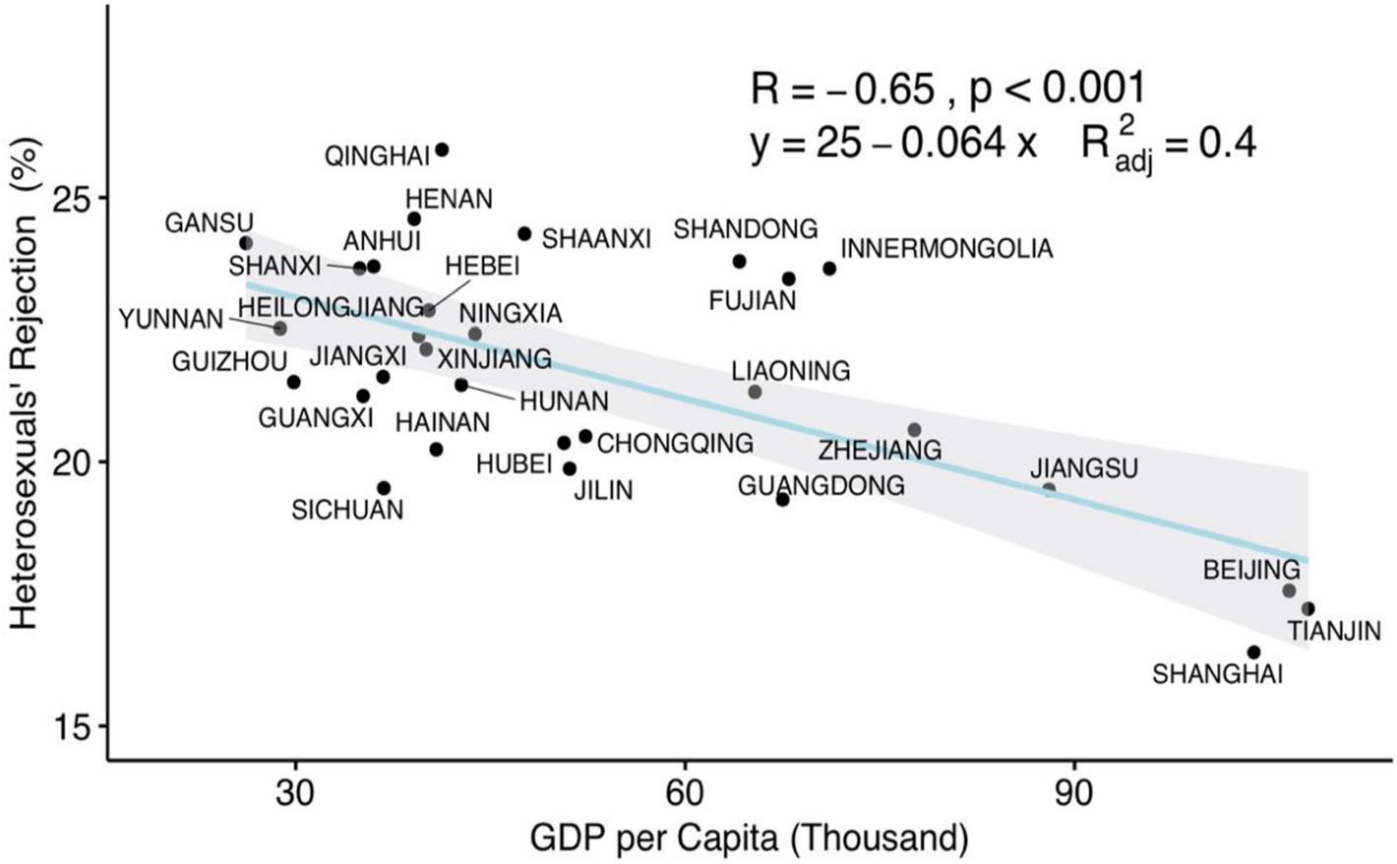
Correlation between GDP per capita and heterosexual participants’ rejection

The severity of reported intolerance towards the LGBT community reported by heterosexual participants was inversely associated with regional GDP. It was found that every 100 thousand RMB increase per capita in GDP lead to a 6.4% decrease in rejection by heterosexuals (Fig. 4).

## Discussion

This is the first Chinese national study which examined discrimination against lesbian, gay, bisexual, and transgender individuals as separate groups. Our results on discrimination were consistent and significantly correlated with those of a previous national study. Between 2014 and 2017, RY Chua, KG Huang and M Jin [22], conducted a study investigating people’s tolerance towards the LGBT community which involved a sample of 11,662 participants across 31 Chinese provinces. We validated these researchers’ previous findings regarding tolerance towards the LGBT community in a larger sample. We also found that the tolerance score in each province perfectly matched that reported by RY Chua, KG Huang and M Jin [22]. They measured tolerance towards the LGBT community from the perspective of provincial residents (e.g., “To what extent are people in your province tolerant towards lesbians, gays, bisexuals and transgender individuals?”) The current study validated their results by directly measuring the attitudes of heterosexual participants. Specifically, the tolerance scores in the current study were calculated from five items, which is a more comprehensive measure than that used in RY Chua, KG Huang and M Jin [22].

In terms of heterosexuals’ acceptance towards the LGBT community, heterosexual participants reported a high level of acceptance of social relationships with LGBT individuals, such as having LGBT friends or colleagues. However, heterosexual participants reported that it was hard to accept their own children identifying as LGBT. This finding is consistent with the self-perceived discrimination in a family setting reported by LGBT participants. For member of the Chinese LGBT community, the greatest source of pressure to conform to societal norms of sexuality and identity comes from family members—particularly parents [9]. The main reason for this might be that Chinese parents have a strong belief in traditional heterosexual marriage, and that they regard marriage as a critical life event and critical aspect of a child’s mandatory duty to carry on the family bloodline [9]. Being LGBT could mean giving up family duty and disappointing parents by not producing offspring [23]. Chinese society places a heavy emphasis on interpersonal relations and community, and Chinese people seldom make individual decisions without evaluating the impact of these decisions on their family [24]. Moreover, based on the cultural expectations of Confucianism, getting married and raising children are considered core values for filial piety [2].

Family acceptance is related to a higher level of self-esteem and positive physical and mental health in LGBT individuals [25]. Chinese LGBT individuals often deal with substantial pressure from their family, instead of receiving their support. It is important to improve the social acceptance of the LGBT community, especially at the family level. It is important to design family interventions to promote parental acceptance of LGBT children in order to improve their health and reduce the pressure they face. Our results show that transgender participants face a greater level of discrimination within their families. Previous research has indicated that family rejection can be predictive of suicide attempts and substance misuse among the transgender population [26]. It is therefore critical to understand the effects of discrimination experienced within families on the mental health of LGBT individuals.

In consistent with previous study in Japan, the current results also showed that LGBT community faced discrimination from various public services settings including the laws and policies [16]. LGBT participants reported severe levels of self-perceived discrimination in social service settings. In China, LGBT couples are not recognized as legal couples with marriage certificates, which consequently leads to a variety of barriers in the context of family law (e.g., inheritance rights, receiving a partner’s life insurance compensation). This could be largely improved with increased support from the government and amendments to existing legislation.

According to Meyer’s minority stress model [27], individuals who are looked down on by society have a higher risk of being discriminated against, which can lead to non-disclosure of their stigmatized status. In China, members of the LGBT community are discriminated against and marginalized by society [18]. As a result, the majority of LGBT individuals tend to conceal their sexual minority identity in order to avoid being discriminated against. The current study found disclosure rates were different for each subgroup, with different disclosure rates in different settings. Of the different settings surveyed, LGBT participants all had the highest disclosure rate in medical service settings, and the lowest disclosure rate in religious group settings. In terms of disclosure to family, we found that less than half (49.4%) of the gay male participants disclosed their sexual orientation to their family members, and the percentages for bisexual, transgender, and lesbian participants were 54.1, 63.8 and 75.1%, respectively. Among the LGBT subgroups, gay male participants were the least likely to disclose to family members, while lesbian participants were the most likely to disclose to family members. The disclosure rates in different settings were consistent with the self-perceived discrimination reported by LGBT participants across those settings. Among the Lesbian, Gay, Bisexual, and Transgender groups, lesbians had the highest disclosure rates in family, school, workplace, and religious groups. This result is consistent with the relatively lower level of self-perceived discrimination reported by lesbian participants compared to gay male, bisexual, and transgender participants.

The results of the present research demonstrate that LGBT individuals in China face discrimination in an array of social settings. Based on the data presented here, we created friendly-hostile maps illustrating the level of discrimination across Chinese provinces. Compared with the economically developed regions (e.g., coastal areas), the economically underdeveloped regions (e.g., north western and central China) showed higher levels of discrimination.

Tolerance towards the LGBT community was positively associated with economic growth [7]. As shown in the friendly-hostile map, LGBT individuals living in areas of lower economic development (e.g., north western) tended to face harsher discrimination. Our findings indicate that higher levels of economic development are associated with a decrease in discrimination towards LGBT individuals. Previous researchers have found that greater inclusion of LGBT individuals in society is positively associated with a country’s economic development, and suggested that LGBT equality should be relevant to economic development programs [7]. The current study confirmed such a relationship, and found that every 100 thousand RMB increase per capita GDP lead to a 6.4% decrease in unacceptable events experienced by LGBT participants. Our findings confirm that economic development is significantly correlated with a decrease in discrimination towards the LGBT community. In addition, substantial evidence from many countries has shown that discrimination and violence against LGBT individuals is harmful to a country’s economy [7]. Reductions in LGBT discrimination and development of the economy are closely linked and can be regarded as harmonious processes.

Furthermore, employment is an important part of the economy. Our results show that transgender and gay male participants are more likely to be unemployed. Organizations should aim to build policies and engage in practices to support LGBT workers. Discrimination in the workplace has negative consequences for workers’ mental and physical health [28]. Organizations have a social responsibility and ethical obligation to provide a friendly and supportive work environment for LGBT workers [28].

There are several limitations of the current study. First, this was a cross-sectional study and causality of the associations cannot be determined. Second, the convenience sampling method used to recruit participants might limit the generalizability of the results. Participants were mainly young people with internet access, and these participants might also be regular users of the LGBT community. Third, we did not cover all aspects of discrimination, for example, views on the legalization of same-sex marriage and child adoption were not assessed. Fourth, measures used in current study were created based on the Chinese cultural context, and the reliability and validity of these measures should be assessed. However, we used expert consultation method to improve the reliability of the measurements in the current study [13, 20, 29]. In future studies, it would be useful to investigate causal relations among perceived discrimination and identifying as LGBT using scales with good psychometric properties.

## Conclusion

In conclusion, this study is an innovative national investigation of the discrimination faced by members of the Chinese LGBT community in various personal and community settings. In order to reduce discrimination, it is important for future studies to discover the underlying reasons for discrimination against LGBT individuals by some heterosexual individuals. Moreover, it is critical for government policies and services to be more tolerant towards the LGBT community, and to provide the legal (e.g., marriage) and medical (e.g., hormone therapy) needs of this community.

## Supplementary information

**Additional file 1.**

## Data Availability

The datasets used and/or analysed during the current study are available from the corresponding author upon request.

## Abbreviations

LGBT: Lesbian, gay, bisexual and transgender
GDP: Gross domestic product
