# Supplementary material for: Mapping out a spectrum of the Chinese public’s discrimination toward the LGBT community: results from a national survey
Source: BMC Public Health. 2020 May 12;20:669. doi: 10.1186/s12889-020-08834-y (PMC7216475; doi:10.1186/s12889-020-08834-y)
Supplement: Supplementary file 1 — Additional file 1. [file 12889_2020_8834_MOESM1_ESM.docx]

Supplementary Information for

Mapping out a spectrum of the Chinese public’s discrimination toward the LGBT community: results from a national survey

**Method**

The inclusion criteria: 1) currently living in Mainland China; 2) the sexual orientation and gender identity of the participant met the definitions used in this study for either lesbian, gay, bisexual, transgender, and heterosexual. In particular, four items were used for the group categorization:

Q1 What is your gender identity and sexual orientation: a) I accept my biological sex and I’m heterosexual; b) I don’t accept my biological sex; c) I’m not heterosexual; d) I don’t accept my biological sex and I’m not heterosexual.

Q2 Which gender do you think you may have romantic feelings towards?: a) Male; b) Female; c) Male and Female; d) All gender identities (male, female, intersex, transgender); e) None; f) I don’t know.

Q3 Which gender do you think you may be sexually attracted to?: a) Male; b) Female; c) Male and Female; d) All gender identities (male, female, intersex, transgender); e) None; f) I don’t know.

Q4 What was your biological sex at birth: a) Male; b) Female; c) Intersex.

According to the corresponding answers, the participants can be categorized into five groups: lesbian, gay, bisexual, transgender, and heterosexual.

The exclusion criteria: 1) intersex; 2) work or get married before 18-year-old; 3) provide obvious unreasonable responses. According to these criteria, 29125 (92.23%) participants were eligible for this study.

## Measures

In particular, the basic characteristics included age, ethnicity, living in urban, highest education level, employment status, religious affiliation, and whether the individual suffered from family violence. For heterosexuals’ acceptance towards LGBT persons and perceived discrimination against the LGBT community, the scores were measured by the ratio of checked options in multiple choice items and by the five (totally negative to totally positive), three (negative, not sure, positive), and two (negative, positive) point scales in other items. All these scores were scaled from 0 to 1 with higher scores indicating greater discrimination or rejection. Specifically, heterosexuals’ acceptance towards LGBT persons was assessed by five items regarding 1) their attitudes towards the LGBT community; 2) positions in their personal sphere they felt it was unacceptable for LGBT persons to occupy (family, roommate, neighbor, leader, colleague, teacher, public figure, others); 3) Their willingness or lack thereof to get close to members of each group (homosexual, bisexual, transgender); 4) Acceptance of sub-group members raising children (homosexual, bisexual, transgender); 5) Acceptance towards their own children claiming to be homosexual, bisexual, or transgender. In addition, the *General rejection* was the averaged discriminatory scores across the five items.

Perceived discrimination against the LGBT community was measured in seven sections representing seven environments (family, media, medical services, religious community, school, social services, and workplace) in which LGBT persons may suffer discrimination. Specifically, discrimination within families was measured by five items: 1) Disclosure of sexual orientation or gender identity to family members (parents or guardian, other senior members, spouse, siblings, junior members); 2) suppression of gender expression; 3) family members’ attitudes towards sexual and gender minorities; 4) marriage and childbearing pressure from family members; 5) sexual or gender based violence events. The average discriminatory scores represented the perceived discrimination in family. Perceived discrimination in the media was measured by seven items. Five point scales were used in these items. Specifically, these items measured how often sexual or gender minority figures or events were seen or heard about in 1) newspapers or magazines, 2) broadcast, television, or movies, 3) internet, and whether the characteristics of these figures or events were presented 1) comprehensively, 2) objectively, 3) in a way that reinforces people’s biases, or 4) promotes equal rights. The weighted average discriminatory scores represented the perceived discrimination in media. The discrimination in medical services was measured by whether patients chose to disclose their sexual orientation or gender identity to doctors, suppressed their gender expression, or experienced any sexual or gender based violence events in medical service locations or mental health clinics. The average discriminatory scores represented the perceived discrimination in medical services. Participants who reported having religious beliefs were asked about discrimination in their religious community. They were asked about the attitude towards sexual minorities in religious doctrines, or those held by the clergy and religious followers, whether they have disclosed their sexual orientation or gender identity to their religious community, whether they have attempted to suppressed their gender expression, and whether they have experienced any sexual or gender based violence events within their religious community. The average discriminatory scores represented the perceived discrimination in religious communities. Discrimination at school was measured by four items querying: 1) gender diversity education; 2) disclosure of sexual orientation or gender identity; 3) suppression of gender expression; 4) sexual or gender based violence events. The average discriminatory scores represented the perceived discrimination at school. Discrimination in social services was measured in three parts: 1) difficulty in accessing different social services (12 items with five point scales, such as applying for social assistance, legal proceedings, pension institutions, gender/name changes, medical insurance); 2) disclosure of sexual orientation or gender identity; 3) suppression of gender expression. The weighted average discriminatory scores represented the perceived discrimination in social service. Participants who work were asked about discrimination in workplaces, which was measured in seven parts: 1) knowledge about sexual and gender minorities in vocational training; 2) written regulations about equal treatment for sexual or gender minorities; 3) disclosure of sexual orientation or gender identity; 4) suppression of gender expression; 5) leaders’ attitudes towards sexual or gender minorities; 6) colleagues’ attitudes towards sexual or gender minorities; 7) sexual or gender based violence events in the workplace. The average discriminatory scores represented perceived discrimination in workplaces. In addition, the average score across the environments indicated the *general discrimination* against the LGBT community.

Figure S1. Sample Categorization procedure


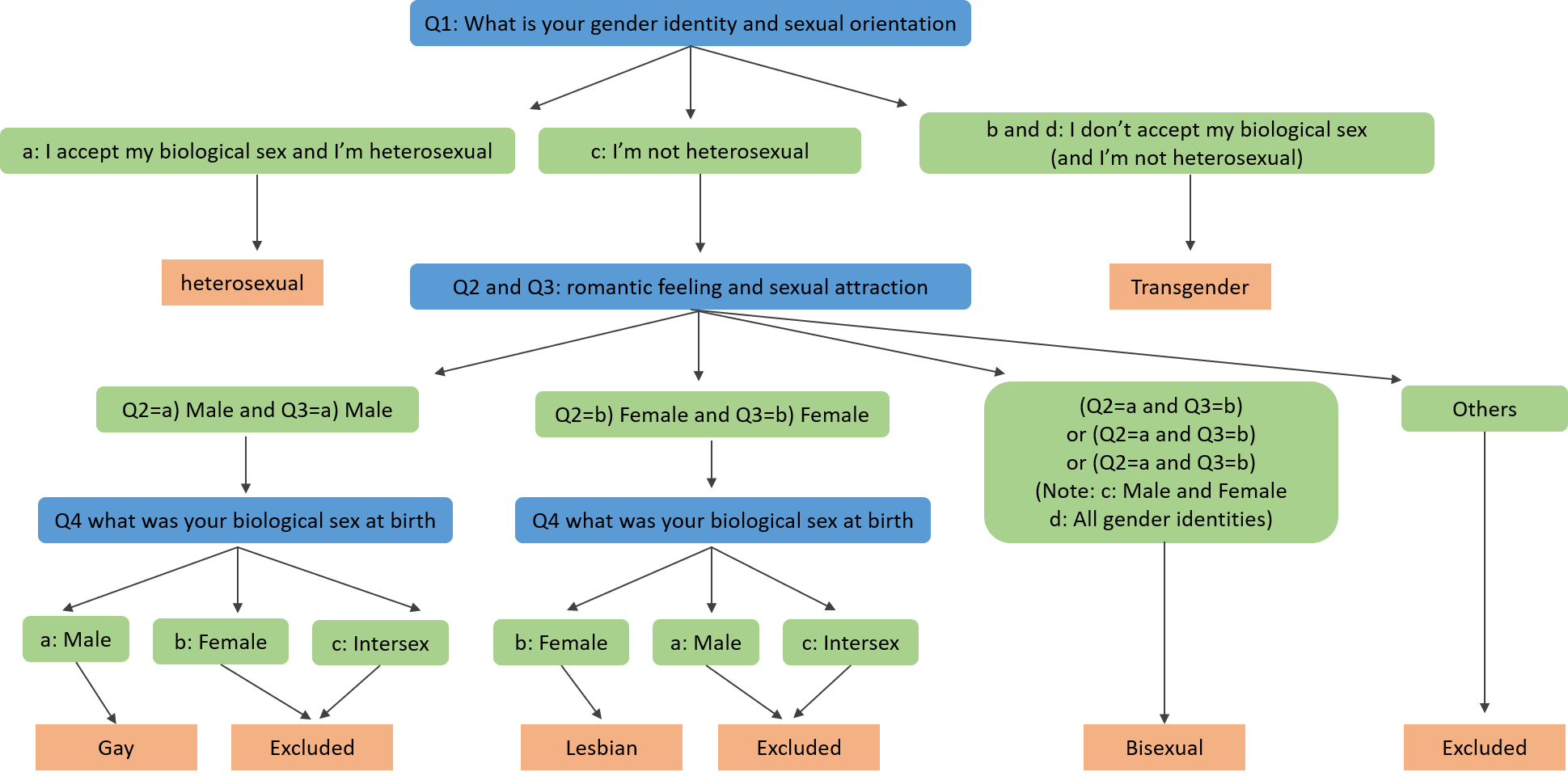


**Figure S2**. Sample size across all the mainland provinces

Table S1. Baseline socio-demographic characteristics of the participants

|  | Lesbian  (n =2066) | | Gay  (n =9491) | | | Bisexual  (n =3441) | | | Transgender  (n =3195) | | | Heterosexual  (n =10932) | | |  |
| --- | --- | --- | --- | --- | --- | --- | --- | --- | --- | --- | --- | --- | --- | --- | --- |
|  | N | % | | N | % | | N | % | | N | % | | N | % | |
| Age <20 | 541 | 26.2 | | 2380 | 25.1 | | 1123 | 32.6 | | 982 | 30.7 | | 2720 | 24.9 | |
| Age 20-29 | 1343 | 65.0 | | 5978 | 63.0 | | 2117 | 61.5 | | 1861 | 58.2 | | 6940 | 63.5 | |
| Age 30-39 | 175 | 8.5 | | 903 | 9.5 | | 166 | 4.8 | | 283 | 8.9 | | 946 | 8.6 | |
| Age 40+ | 7 | 0.3 | | 230 | 2.4 | | 35 | 1.0 | | 69 | 2.2 | | 326 | 3.0 | |
| Han Chinese | 1910 | 92.5 | | 8818 | 92.9 | | 3190 | 92.7 | | 2946 | 92.2 | | 10194 | 93.3 | |
| Urban residency | 2027 | 98.1 | | 9014 | 95.0 | | 3359 | 97.6 | | 2952 | 92.4 | | 10415 | 95.3 | |
| Attended college or above | 1253 | 60.7 | | 5169 | 54.5 | | 2218 | 64.5 | | 1225 | 38.3 | | 6206 | 56.8 | |
| Unemployed | 198 | 9.6 | | 996 | 10.5 | | 265 | 7.7 | | 450 | 14.1 | | 1113 | 10.2 | |
| Having religious group | 296 | 14.3 | | 1672 | 17.6 | | 484 | 14.1 | | 705 | 22.1 | | 1809 | 16.6 | |
| Having disability | 9 | 0.4 | | 95 | 1.0 | | 26 | 0.8 | | 41 | 1.3 | | - | - | |
| Married: | 51 | 2.5 | | 436 | 4.6 | | 164 | 4.8 | | 228 | 7.1 | | - | - | |
| Heterosexual Marriage | 19 | 37.3 | | 377 | 86.5 | | 151 | 92.1 | | 201 | 88.2 | | - | - | |
| Cooperative marriage | 30 | 58.8 | | 52 | 11.9 | | 13 | 7.9 | | 23 | 10.1 | | - | - | |
| Married oversea | 2 | 3.9 | | 7 | 1.6 | | 0 | 0 | | 4 | 1.8 | | - | - | |
